# Where Is the Responsibility?

**Published:** 1886-04-20

**Authors:** 


### WHERE IS THE RESPONSIBILITY?

The delicate nature of criminal ab[illegible]rtion proves a barrier to the discussion of its hideous features through the columns of our newspapers, but it behooves every honest man and woman in our community to understand the bearing of such proceedings upon the welfare of society.

The fatal result of violence supposed to have been resorted to for the accomplishment of abortion in a case which has come before the criminal court of Fulton County, demands investigation in a way to find out the source of such violent means towards attaining this end. That a woman should come from another State, under an assumed name, to seek professional assistance, affords prima facia evidence of an illicit object, and that this individual, who is represented to be entirely without pecuniary resources, should be provided with board ing and lodging, with a nurse, while able to move about the house, raises a grave question as to the nature of the case, and a knowledge of the facts, on the part of her medical attendant. His innocence is sought to be shown by the statement of a colleague who was called in when the patient was about to die—that the declaration was made to him as to the fact of an abortion having occurred, but nothing is learned in regard to the means by which it was accomplished. Another plausible explanation is made, that the signs of injury to the interior structure of the womb may have resulted from the use of a silver female catheter for washing out the cavity, in accordance with the advice of the said colleague. A still more complete exhoneration of the attending physician is based upon the assertion of a medical man, who was present at the post mortem examination, that there was no indications of the use of any instrument whatever or other signs of violence to the womb. On the other hand, the representations of other competent members of the medical profession, who witnessed the same examination, were to the effect that violent measures had been resorted to during the life of the patient, presumably to secure the expulsion of the fœtus from the womb. The statement of other physicians and surgeons of recognized capacity and practical acquaintance with such pathological specimens, upon examination of the lesions in the uterine structure, confirmed the observation of those who had inferred that death ensued from violence to the womb, which was extended to the lining membrane of the abdomen in the form of peritonitis, and propagated through the nervous system to the brain.

The matter of responsibility for resorting to such violence as was proven to exist by ample medical testimony, rests either upon the woman herself, or upon some accomplice in this undertaking, and every good citizen would doubtless rejoice to have the question relieved of the doubts which are thrown around it by the verdict of the coroner's inquest; so that it was respectfully submitted as a proper subject for the investigation of our Grand Jury, and a true bill was found against Dr. E. H. Green, who had been in charge of the patient.

